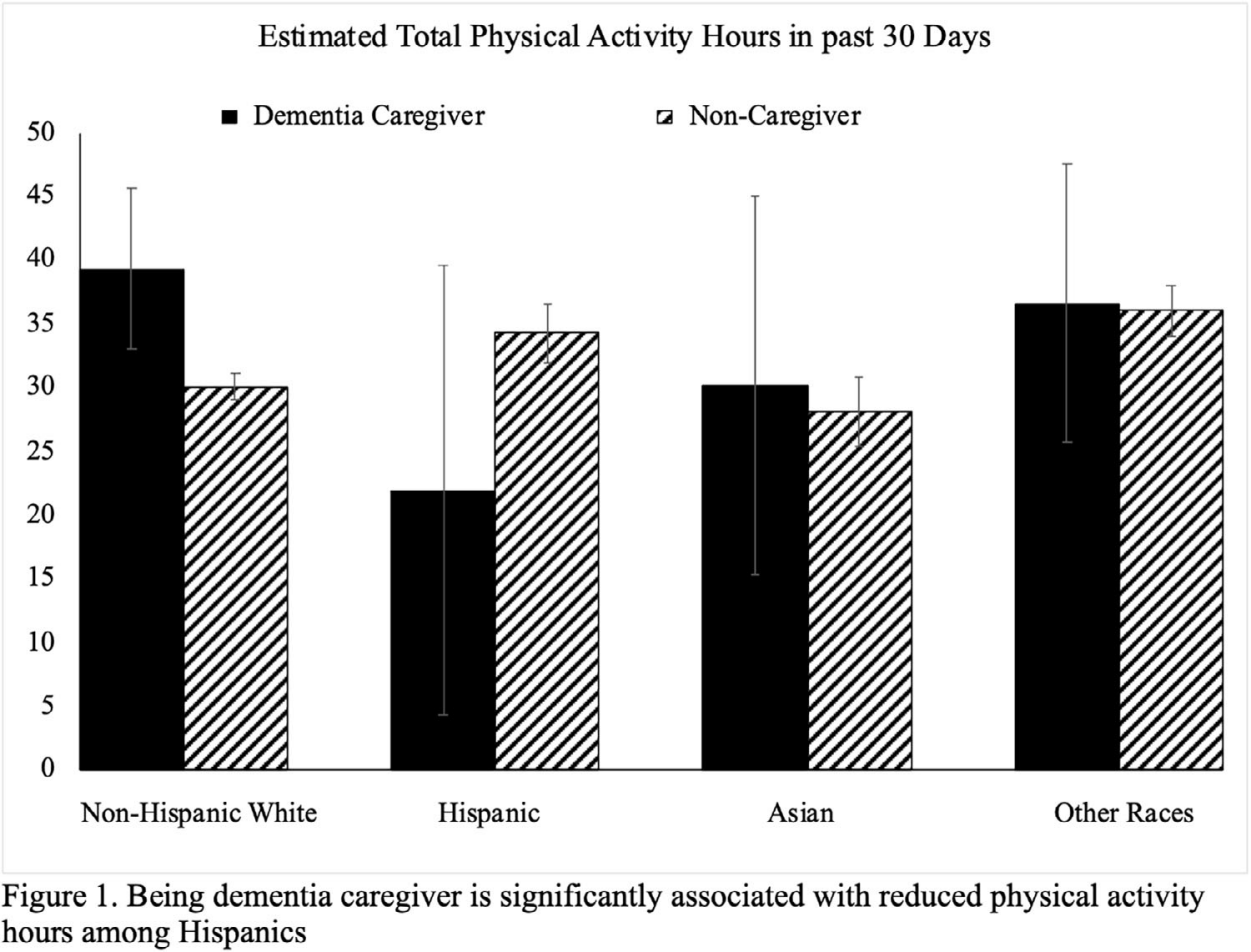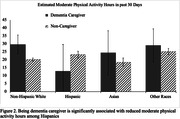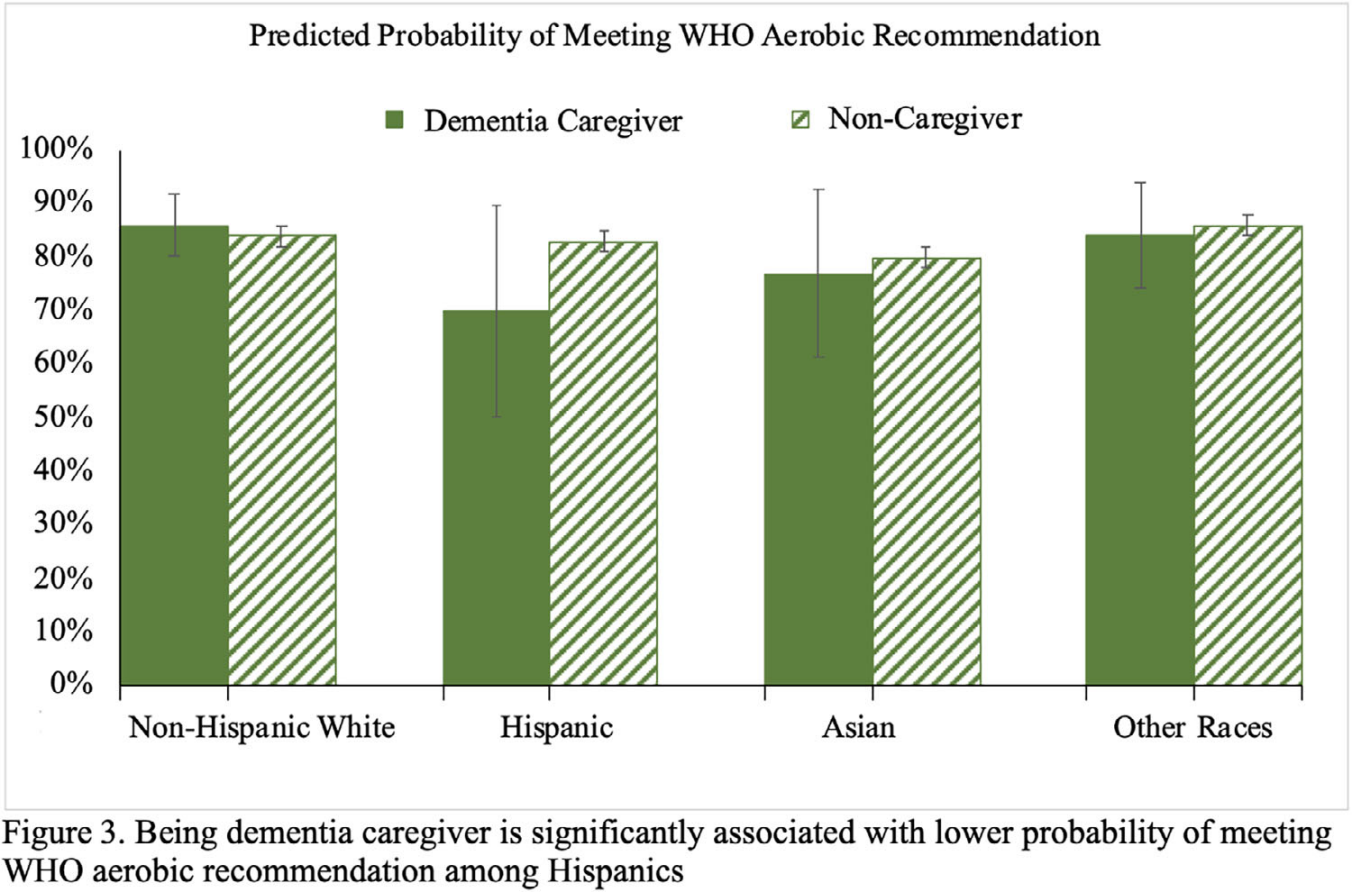# Racial/Ethnic Differences in Physical Activity Participation of Dementia Caregivers in the US: Findings from the 2017 BRFSS Survey

**DOI:** 10.1002/alz.085156

**Published:** 2025-01-09

**Authors:** Jiaming Liang

**Affiliations:** ^1^ USC Edward R. Roybal Institute on Aging, University of Southern California, Los Angeles, CA USA

## Abstract

**Background:**

It is well documented that participating in physical activity can help dementia caregivers alleviate stress and enhance well‐being. However, few studies have examined dementia caregivers’ needs for exercise, and the feasibility of promoting their physical activity amidst heavy caregiving responsibilities. This study compared the participation of physical activity between dementia caregivers and non‐caregivers, and examined effects of racial/ethnic identities and other sociodemographic factors on dementia caregivers’ physical activity participation.

**Method:**

A cross‐sectional study was conducted using the 2017 Behavioral Risk Factor Surveillance System (BRFSS) Survey. Participants’ physical activity engagement was measured using four indicators: hours of (1) all physical activities, (2) moderate activities, and (3) vigorous activities in the last 30 days, and (4) whether meeting the WHO aerobic exercise recommendation. Regression and interaction analyses were used to examine associations between being dementia caregivers and physical activity participation, and factors related to physical activity status of dementia caregivers.

**Result:**

The study included 10,158 participants (280 dementia caregivers and 9,878 non‐caregivers). Compared to non‐caregivers, dementia caregivers tend to be older (≥ 55‐year‐old, 67% vs. 55%), more likely to be women (63% vs. 51%), have a higher level of education (≥ Bachelor’s degree, 56% vs. 50%), and more likely to own houses (83% vs. 71%). Between‐group comparison found no difference between dementia caregivers and non‐caregivers on all four physical activity indicators. Regression analyses found that, Hispanic participants tend to report more hours of physical activity participation than non‐Hispanic Whites. However, interaction analyses revealed that Hispanic dementia caregivers have significantly fewer hours of participating in moderate physical activity (12.96±8.52 vs 23.20±1.10), and are less likely to meet the WHO aerobic recommendation (OR: 0.70±0.10 vs 0.83± 0.10) than Hispanic non‐caregivers. The analyses also found that dementia caregivers report fewer moderate activity hours if they are unemployed (15.71±5.92 vs. 23.44±0.99).

**Conclusion:**

The findings suggest that dementia caregiving does not necessarily reduce physical activity hours of caregivers, unless they are Hispanic and unemployed. Future intervention development should focus on promoting moderate and aerobic exercise participation among dementia caregivers, especially in developing culturally and linguistically appropriate programs for Hispanic dementia caregivers.